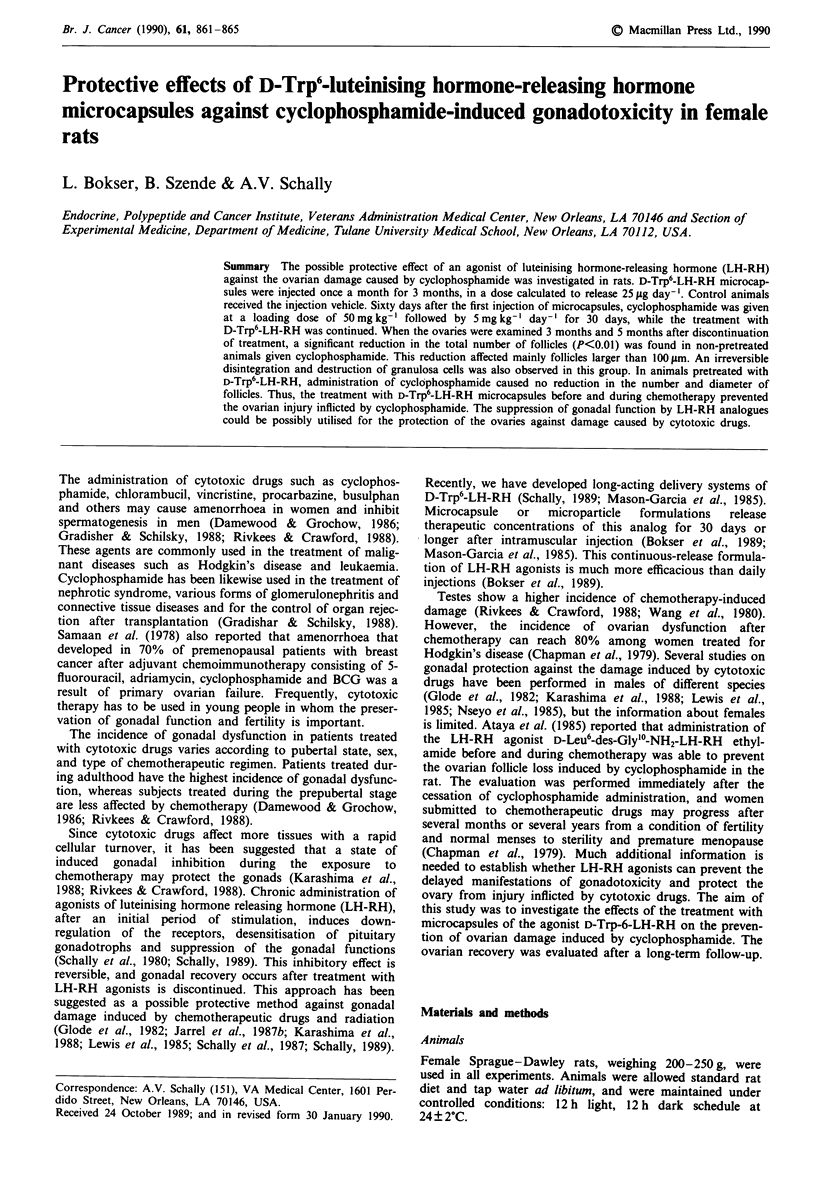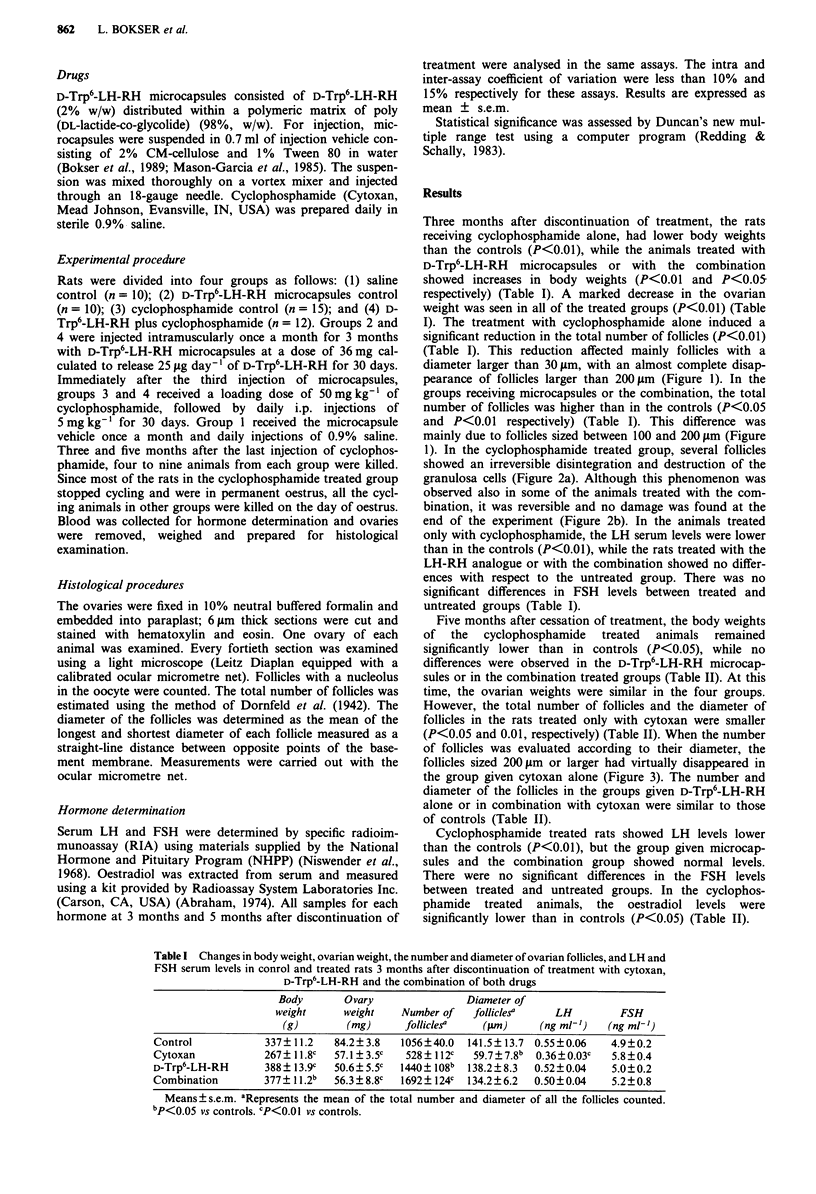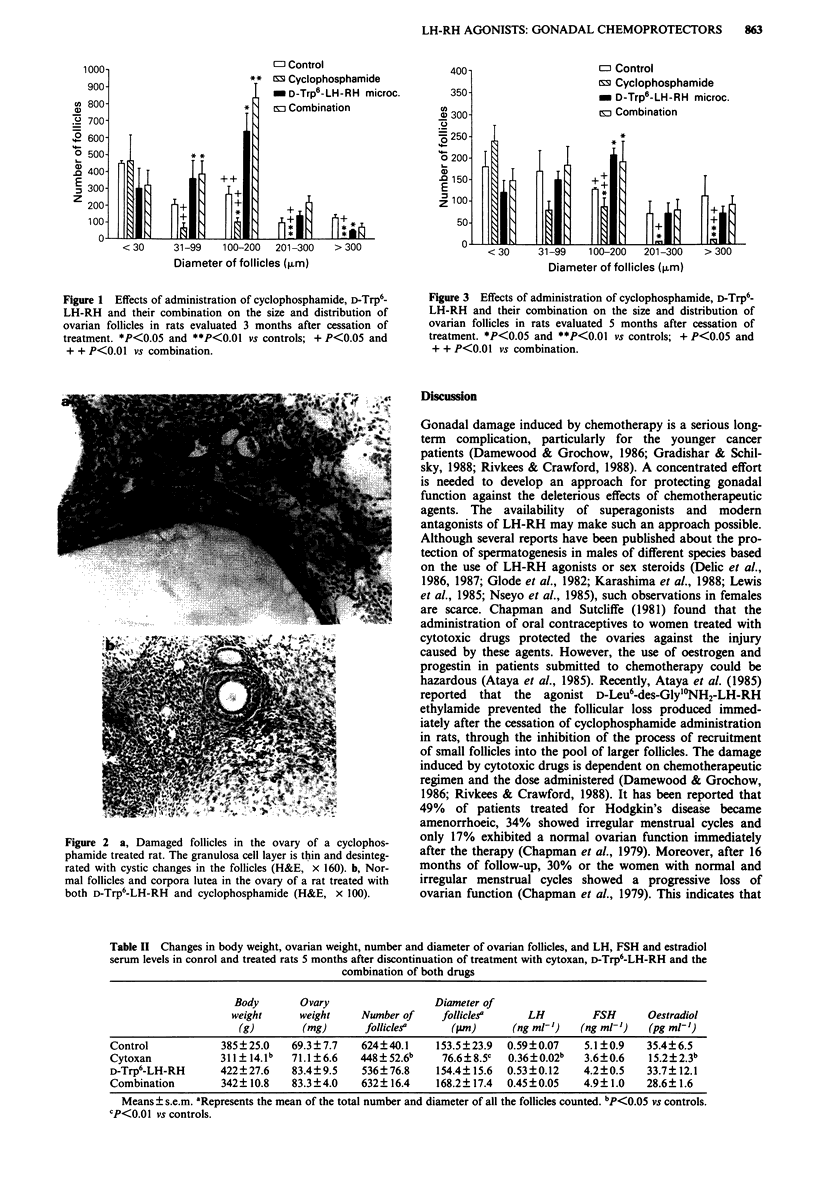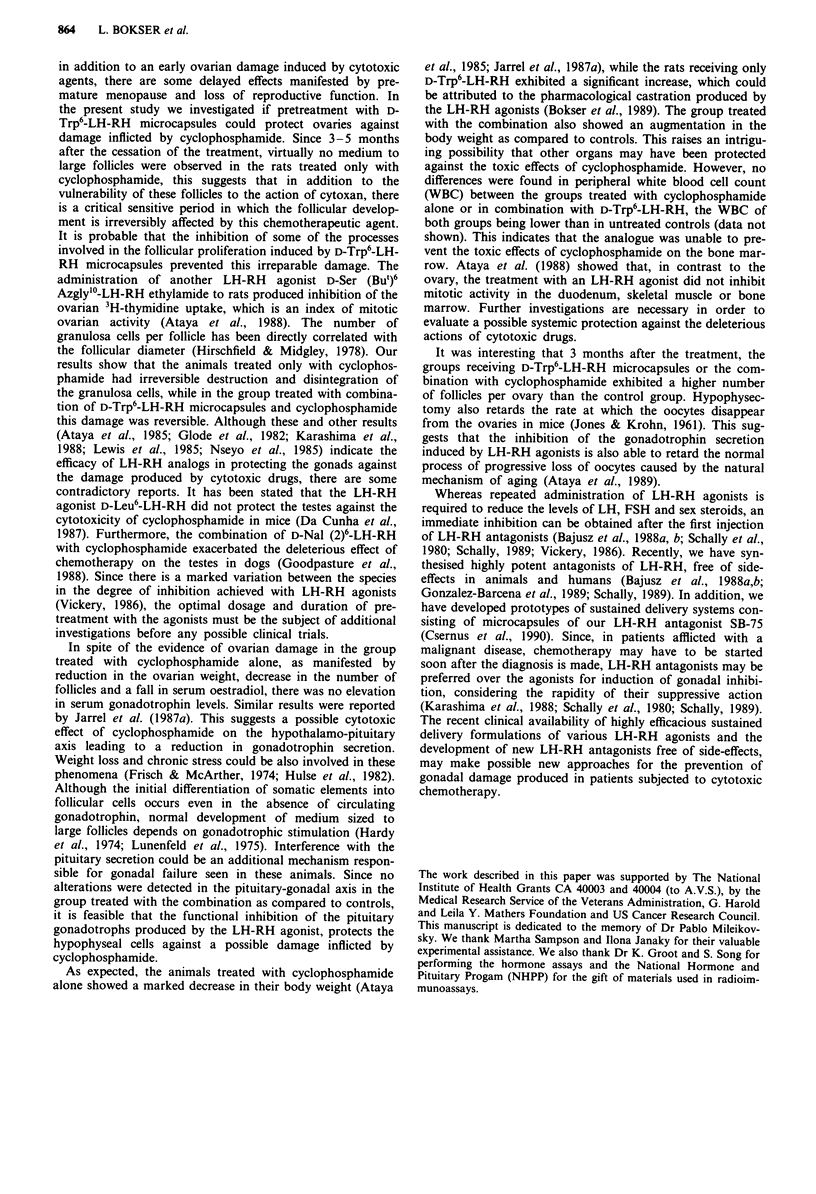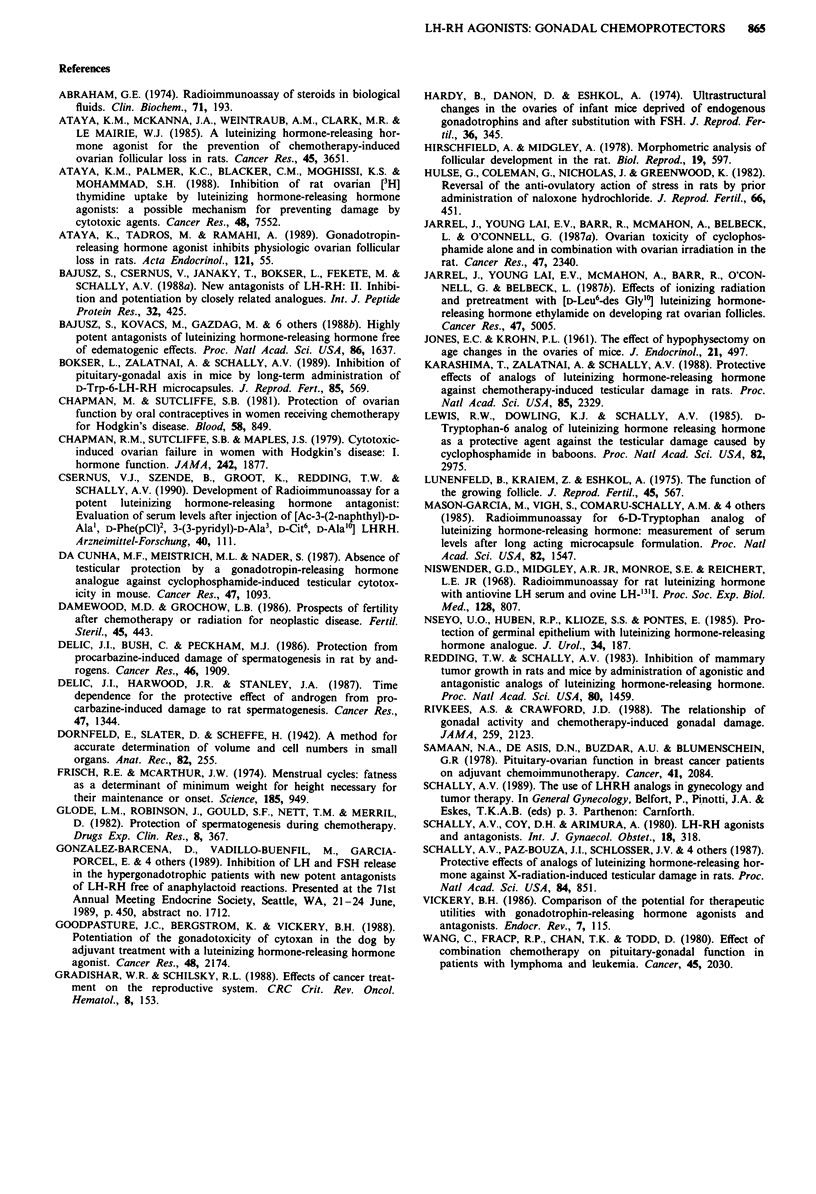# Protective effects of D-Trp6-luteinising hormone-releasing hormone microcapsules against cyclophosphamide-induced gonadotoxicity in female rats.

**DOI:** 10.1038/bjc.1990.192

**Published:** 1990-06

**Authors:** L. Bokser, B. Szende, A. V. Schally

**Affiliations:** Endocrine, Polypeptide and Cancer Institute, Veterans Administration Medical Center, New Orleans, LA 70146.

## Abstract

**Images:**


Br. J. Cancer (1990), 61, 861-865 © Macmillan Press Ltd., 1990

### Protective effects of D-Trp[6]-luteinising hormone-releasing hormone microcapsules against cyclophosphamide-induced gonadotoxicity in female rats

L. Bokser, B. Szende & A.V. Schally

*Endocrine, Polypeptide and Cancer Institute, Veterans Administration Medical Center, New Orleans, LA 70146 and Section of Experimental Medicine, Department of Medicine, Tulane University Medical School, New Orleans, LA 70112, USA.*

**Summary** The possible protective effect of an agonist of luteinising hormone-releasing hormone (LH-RH) against the ovarian damage caused by cyclophosphamide was investigated in rats. D-Trp[6]-LH-RH microcapsules were injected once a month for 3 months, in a dose calculated to release 25 $\mu g$ $day^{-1}$. Control animals received the injection vehicle. Sixty days after the first injection of microcapsules, cyclophosphamide was given at a loading dose of 50 mg $kg^{-1}$ followed by 5 mg $kg^{-1}$ $day^{-1}$ for 30 days, while the treatment with D-Trp[6]-LH-RH was continued. When the ovaries were examined 3 months and 5 months after discontinuation of treatment, a significant reduction in the total number of follicles ($P<0.01$) was found in non-pretreated animals given cyclophosphamide. This reduction affected mainly follicles larger than 100 $\mu m$. An irreversible disintegration and destruction of granulosa cells was also observed in this group. In animals pretreated with D-Trp[6]-LH-RH, administration of cyclophosphamide caused no reduction in the number and diameter of follicles. Thus, the treatment with D-Trp[6]-LH-RH microcapsules before and during chemotherapy prevented the ovarian injury inflicted by cyclophosphamide. The suppression of gonadal function by LH-RH analogues could be possibly utilised for the protection of the ovaries against damage caused by cytotoxic drugs.

The administration of cytotoxic drugs such as cyclophosphamide, chlorambucil, vincristine, procarbazine, busulphan and others may cause amenorrhoea in women and inhibit spermatogenesis in men (Damewood & Grochow, 1986; Gradisher & Schilsky, 1988; Rivkees & Crawford, 1988). These agents are commonly used in the treatment of malignant diseases such as Hodgkin's disease and leukaemia. Cyclophosphamide has been likewise used in the treatment of nephrotic syndrome, various forms of glomerulonephritis and connective tissue diseases and for the control of organ rejection after transplantation (Gradishar & Schilsky, 1988). Samaan *et al.* (1978) also reported that amenorrhoea that developed in 70% of premenopausal patients with breast cancer after adjuvant chemoimmunotherapy consisting of 5-fluorouracil, adriamycin, cyclophosphamide and BCG was a result of primary ovarian failure. Frequently, cytotoxic therapy has to be used in young people in whom the preservation of gonadal function and fertility is important.

The incidence of gonadal dysfunction in patients treated with cytotoxic drugs varies according to pubertal state, sex, and type of chemotherapeutic regimen. Patients treated during adulthood have the highest incidence of gonadal dysfunction, whereas subjects treated during the prepubertal stage are less affected by chemotherapy (Damewood & Grochow, 1986; Rivkees & Crawford, 1988).

Since cytotoxic drugs affect more tissues with a rapid cellular turnover, it has been suggested that a state of induced gonadal inhibition during the exposure to chemotherapy may protect the gonads (Karashima *et al.*, 1988; Rivkees & Crawford, 1988). Chronic administration of agonists of luteinising hormone releasing hormone (LH-RH), after an initial period of stimulation, induces down-regulation of the receptors, desensitisation of pituitary gonadotrophs and suppression of the gonadal functions (Schally *et al.*, 1980; Schally, 1989). This inhibitory effect is reversible, and gonadal recovery occurs after treatment with LH-RH agonists is discontinued. This approach has been suggested as a possible protective method against gonadal damage induced by chemotherapeutic drugs and radiation (Glode *et al.*, 1982; Jarrel *et al.*, 1987*b*; Karashima *et al.*, 1988; Lewis *et al.*, 1985; Schally *et al.*, 1987; Schally, 1989).

Recently, we have developed long-acting delivery systems of D-Trp[6]-LH-RH (Schally, 1989; Mason-Garcia *et al.*, 1985). Microcapsule or microparticle formulations release therapeutic concentrations of this analog for 30 days or longer after intramuscular injection (Bokser *et al.*, 1989; Mason-Garcia *et al.*, 1985). This continuous-release formulation of LH-RH agonists is much more efficacious than daily injections (Bokser *et al.*, 1989).

Testes show a higher incidence of chemotherapy-induced damage (Rivkees & Crawford, 1988; Wang *et al.*, 1980). However, the incidence of ovarian dysfunction after chemotherapy can reach 80% among women treated for Hodgkin's disease (Chapman *et al.*, 1979). Several studies on gonadal protection against the damage induced by cytotoxic drugs have been performed in males of different species (Glode *et al.*, 1982; Karashima *et al.*, 1988; Lewis *et al.*, 1985; Nseyo *et al.*, 1985), but the information about females is limited. Ataya *et al.* (1985) reported that administration of the LH-RH agonist D-Leu[6]-des-Gly[10]-$NH_2$-LH-RH ethylamide before and during chemotherapy was able to prevent the ovarian follicle loss induced by cyclophosphamide in the rat. The evaluation was performed immediately after the cessation of cyclophosphamide administration, and women submitted to chemotherapeutic drugs may progress after several months or several years from a condition of fertility and normal menses to sterility and premature menopause (Chapman *et al.*, 1979). Much additional information is needed to establish whether LH-RH agonists can prevent the delayed manifestations of gonadotoxicity and protect the ovary from injury inflicted by cytotoxic drugs. The aim of this study was to investigate the effects of the treatment with microcapsules of the agonist D-Trp-6-LH-RH on the prevention of ovarian damage induced by cyclophosphamide. The ovarian recovery was evaluated after a long-term follow-up.

### Materials and methods

### *Animals*

Female Sprague-Dawley rats, weighing 200-250 g, were used in all experiments. Animals were allowed standard rat diet and tap water *ad libitum*, and were maintained under controlled conditions: 12 h light, 12 h dark schedule at 24±2°C.

Correspondence: A.V. Schally (151), VA Medical Center, 1601 Perdido Street, New Orleans, LA 70146, USA.
Received 24 October 1989; and in revised form 30 January 1990.

### Drugs

D-Trp[6]-LH-RH microcapsules consisted of D-Trp[6]-LH-RH (2% w/w) distributed within a polymeric matrix of poly (DL-lactide-co-glycolide) (98%, w/w). For injection, microcapsules were suspended in 0.7 ml of injection vehicle consisting of 2% CM-cellulose and 1% Tween 80 in water (Bokser *et al.*, 1989; Mason-Garcia *et al.*, 1985). The suspension was mixed thoroughly on a vortex mixer and injected through an 18-gauge needle. Cyclophosphamide (Cytoxan, Mead Johnson, Evansville, IN, USA) was prepared daily in sterile 0.9% saline.

### Experimental procedure

Rats were divided into four groups as follows: (1) saline control ($n = 10$); (2) D-Trp[6]-LH-RH microcapsules control ($n = 10$); (3) cyclophosphamide control ($n = 15$); and (4) D-Trp[6]-LH-RH plus cyclophosphamide ($n = 12$). Groups 2 and 4 were injected intramuscularly once a month for 3 months with D-Trp[6]-LH-RH microcapsules at a dose of 36 mg calculated to release 25 $\mu g\ day^{-1}$ of D-Trp[6]-LH-RH for 30 days. Immediately after the third injection of microcapsules, groups 3 and 4 received a loading dose of 50 $mg\ kg^{-1}$ of cyclophosphamide, followed by daily i.p. injections of 5 $mg\ kg^{-1}$ for 30 days. Group 1 received the microcapsule vehicle once a month and daily injections of 0.9% saline. Three and five months after the last injection of cyclophosphamide, four to nine animals from each group were killed. Since most of the rats in the cyclophosphamide treated group stopped cycling and were in permanent oestrus, all the cycling animals in other groups were killed on the day of oestrus. Blood was collected for hormone determination and ovaries were removed, weighed and prepared for histological examination.

### Histological procedures

The ovaries were fixed in 10% neutral buffered formalin and embedded into paraplast; 6 $\mu m$ thick sections were cut and stained with hematoxylin and eosin. One ovary of each animal was examined. Every fortieth section was examined using a light microscope (Leitz Diaplan equipped with a calibrated ocular micrometre net). Follicles with a nucleolus in the oocyte were counted. The total number of follicles was estimated using the method of Dornfeld *et al.* (1942). The diameter of the follicles was determined as the mean of the longest and shortest diameter of each follicle measured as a straight-line distance between opposite points of the basement membrane. Measurements were carried out with the ocular micrometre net.

### Hormone determination

Serum LH and FSH were determined by specific radioimmunoassay (RIA) using materials supplied by the National Hormone and Pituitary Program (NHPP) (Niswender *et al.*, 1968). Oestradiol was extracted from serum and measured using a kit provided by Radioassay System Laboratories Inc. (Carson, CA, USA) (Abraham, 1974). All samples for each hormone at 3 months and 5 months after discontinuation of treatment were analysed in the same assays. The intra and inter-assay coefficient of variation were less than 10% and 15% respectively for these assays. Results are expressed as mean ± s.e.m.

Statistical significance was assessed by Duncan's new multiple range test using a computer program (Redding & Schally, 1983).

### Results

Three months after discontinuation of treatment, the rats receiving cyclophosphamide alone, had lower body weights than the controls ($P<0.01$), while the animals treated with D-Trp[6]-LH-RH microcapsules or with the combination showed increases in body weights ($P<0.01$ and $P<0.05$ respectively) (Table I). A marked decrease in the ovarian weight was seen in all of the treated groups ($P<0.01$) (Table I). The treatment with cyclophosphamide alone induced a significant reduction in the total number of follicles ($P<0.01$) (Table I). This reduction affected mainly follicles with a diameter larger than 30 $\mu m$, with an almost complete disappearance of follicles larger than 200 $\mu m$ (Figure 1). In the groups receiving microcapsules or the combination, the total number of follicles was higher than in the controls ($P<0.05$ and $P<0.01$ respectively) (Table I). This difference was mainly due to follicles sized between 100 and 200 $\mu m$ (Figure 1). In the cyclophosphamide treated group, several follicles showed an irreversible disintegration and destruction of the granulosa cells (Figure 2a). Although this phenomenon was observed also in some of the animals treated with the combination, it was reversible and no damage was found at the end of the experiment (Figure 2b). In the animals treated only with cyclophosphamide, the LH serum levels were lower than in the controls ($P<0.01$), while the rats treated with the LH-RH analogue or with the combination showed no differences with respect to the untreated group. There was no significant differences in FSH levels between treated and untreated groups (Table I).

Five months after cessation of treatment, the body weights of the cyclophosphamide treated animals remained significantly lower than in controls ($P<0.05$), while no differences were observed in the D-Trp[6]-LH-RH microcapsules or in the combination treated groups (Table II). At this time, the ovarian weights were similar in the four groups. However, the total number of follicles and the diameter of follicles in the rats treated only with cytoxan were smaller ($P<0.05$ and 0.01, respectively) (Table II). When the number of follicles was evaluated according to their diameter, the follicles sized 200 $\mu m$ or larger had virtually disappeared in the group given cytoxan alone (Figure 3). The number and diameter of the follicles in the groups given D-Trp[6]-LH-RH alone or in combination with cytoxan were similar to those of controls (Table II).

Cyclophosphamide treated rats showed LH levels lower than the controls ($P<0.01$), but the group given microcapsules and the combination group showed normal levels. There were no significant differences in the FSH levels between treated and untreated groups. In the cyclophosphamide treated animals, the oestradiol levels were significantly lower than in controls ($P<0.05$) (Table II).

**Table I** Changes in body weight, ovarian weight, the number and diameter of ovarian follicles, and LH and FSH serum levels in conrol and treated rats 3 months after discontinuation of treatment with cytoxan, D-Trp[6]-LH-RH and the combination of both drugs

| | *Body weight (g)* | *Ovary weight (mg)* | *Number of follicles*[a] | *Diameter of follicles*[a] *(μm)* | *LH (ng ml⁻¹)* | *FSH (ng ml⁻¹)* |
|---|---|---|---|---|---|---|
| Control | 337±11.2 | 84.2±3.8 | 1056±40.0 | 141.5±13.7 | 0.55±0.06 | 4.9±0.2 |
| Cytoxan | 267±11.8[c] | 57.1±3.5[c] | 528±112[c] | 59.7±7.8[b] | 0.36±0.03[c] | 5.8±0.4 |
| D-Trp[6]-LH-RH | 388±13.9[c] | 50.6±5.5[c] | 1440±108[b] | 138.2±8.3 | 0.52±0.04 | 5.0±0.2 |
| Combination | 377±11.2[b] | 56.3±8.8[c] | 1692±124[c] | 134.2±6.2 | 0.50±0.04 | 5.2±0.8 |

Means±s.e.m. [a]Represents the mean of the total number and diameter of all the follicles counted. [b]$P<0.05$ *vs* controls. [c]$P<0.01$ *vs* controls.

Figure 1 Effects of administration of cyclophosphamide, D-Trp$^6$-LH-RH and their combination on the size and distribution of ovarian follicles in rats evaluated 3 months after cessation of treatment. *$P<0.05$ and **$P<0.01$ *vs* controls; + $P<0.05$ and + + $P<0.01$ *vs* combination.

Figure 3 Effects of administration of cyclophosphamide, D-Trp$^6$-LH-RH and their combination on the size and distribution of ovarian follicles in rats evaluated 5 months after cessation of treatment. *$P<0.05$ and **$P<0.01$ *vs* controls; + $P<0.05$ and + + $P<0.01$ *vs* combination.

Figure 2 **a**, Damaged follicles in the ovary of a cyclophosphamide treated rat. The granulosa cell layer is thin and desintegrated with cystic changes in the follicles (H&E, × 160). **b**, Normal follicles and corpora lutea in the ovary of a rat treated with both D-Trp$^6$-LH-RH and cyclophosphamide (H&E, × 100).

### Discussion

Gonadal damage induced by chemotherapy is a serious long-term complication, particularly for the younger cancer patients (Damewood & Grochow, 1986; Gradishar & Schilsky, 1988; Rivkees & Crawford, 1988). A concentrated effort is needed to develop an approach for protecting gonadal function against the deleterious effects of chemotherapeutic agents. The availability of superagonists and modern antagonists of LH-RH may make such an approach possible. Although several reports have been published about the protection of spermatogenesis in males of different species based on the use of LH-RH agonists or sex steroids (Delic *et al.*, 1986, 1987; Glode *et al.*, 1982; Karashima *et al.*, 1988; Lewis *et al.*, 1985; Nseyo *et al.*, 1985), such observations in females are scarce. Chapman and Sutcliffe (1981) found that the administration of oral contraceptives to women treated with cytotoxic drugs protected the ovaries against the injury caused by these agents. However, the use of oestrogen and progestin in patients submitted to chemotherapy could be hazardous (Ataya *et al.*, 1985). Recently, Ataya *et al.* (1985) reported that the agonist D-Leu$^6$-des-Gly$^{10}$NH$_2$-LH-RH ethylamide prevented the follicular loss produced immediately after the cessation of cyclophosphamide administration in rats, through the inhibition of the process of recruitment of small follicles into the pool of larger follicles. The damage induced by cytotoxic drugs is dependent on chemotherapeutic regimen and the dose administered (Damewood & Grochow, 1986; Rivkees & Crawford, 1988). It has been reported that 49% of patients treated for Hodgkin's disease became amenorrhoeic, 34% showed irregular menstrual cycles and only 17% exhibited a normal ovarian function immediately after the therapy (Chapman *et al.*, 1979). Moreover, after 16 months of follow-up, 30% or the women with normal and irregular menstrual cycles showed a progressive loss of ovarian function (Chapman *et al.*, 1979). This indicates that

**Table II** Changes in body weight, ovarian weight, number and diameter of ovarian follicles, and LH, FSH and estradiol serum levels in conrol and treated rats 5 months after discontinuation of treatment with cytoxan, D-Trp$^6$-LH-RH and the combination of both drugs

| | *Body weight (g)* | *Ovary weight (mg)* | *Number of follicles*$^a$ | *Diameter of follicles*$^a$ *(μm)* | *LH (ng ml$^{-1}$)* | *FSH (ng ml$^{-1}$)* | *Oestradiol (pg ml$^{-1}$)* |
|---|---|---|---|---|---|---|---|
| Control | 385±25.0 | 69.3±7.7 | 624±40.1 | 153.5±23.9 | 0.59±0.07 | 5.1±0.9 | 35.4±6.5 |
| Cytoxan | 311±14.1$^b$ | 71.1±6.6 | 448±52.6$^b$ | 76.6±8.5$^c$ | 0.36±0.02$^b$ | 3.6±0.6 | 15.2±2.3$^b$ |
| D-Trp$^6$-LH-RH | 422±27.6 | 83.4±9.5 | 536±76.8 | 154.4±15.6 | 0.53±0.12 | 4.2±0.5 | 33.7±12.1 |
| Combination | 342±10.8 | 83.3±4.0 | 632±16.4 | 168.2±17.4 | 0.45±0.05 | 4.9±1.0 | 28.6±1.6 |

Means±s.e.m. $^a$Represents the mean of the total number and diameter of all the follicles counted. $^b P<0.05$ *vs* controls. $^c P<0.01$ *vs* controls.

in addition to an early ovarian damage induced by cytotoxic agents, there are some delayed effects manifested by premature menopause and loss of reproductive function. In the present study we investigated if pretreatment with D-Trp$^6$-LH-RH microcapsules could protect ovaries against damage inflicted by cyclophosphamide. Since 3–5 months after the cessation of the treatment, virtually no medium to large follicles were observed in the rats treated only with cyclophosphamide, this suggests that in addition to the vulnerability of these follicles to the action of cytoxan, there is a critical sensitive period in which the follicular development is irreversibly affected by this chemotherapeutic agent. It is probable that the inhibition of some of the processes involved in the follicular proliferation induced by D-Trp$^6$-LH-RH microcapsules prevented this irreparable damage. The administration of another LH-RH agonist D-Ser (Bu$^t$)$^6$ Azgly$^{10}$-LH-RH ethylamide to rats produced inhibition of the ovarian $^3$H-thymidine uptake, which is an index of mitotic ovarian activity (Ataya *et al.*, 1988). The number of granulosa cells per follicle has been directly correlated with the follicular diameter (Hirschfield & Midgley, 1978). Our results show that the animals treated only with cyclophosphamide had irreversible destruction and disintegration of the granulosa cells, while in the group treated with combination of D-Trp$^6$-LH-RH microcapsules and cyclophosphamide this damage was reversible. Although these and other results (Ataya *et al.*, 1985; Glode *et al.*, 1982; Karashima *et al.*, 1988; Lewis *et al.*, 1985; Nseyo *et al.*, 1985) indicate the efficacy of LH-RH analogs in protecting the gonads against the damage produced by cytotoxic drugs, there are some contradictory reports. It has been stated that the LH-RH agonist D-Leu$^6$-LH-RH did not protect the testes against the cytotoxicity of cyclophosphamide in mice (Da Cunha *et al.*, 1987). Furthermore, the combination of D-Nal (2)$^6$-LH-RH with cyclophosphamide exacerbated the deleterious effect of chemotherapy on the testes in dogs (Goodpasture *et al.*, 1988). Since there is a marked variation between the species in the degree of inhibition achieved with LH-RH agonists (Vickery, 1986), the optimal dosage and duration of pretreatment with the agonists must be the subject of additional investigations before any possible clinical trials.

In spite of the evidence of ovarian damage in the group treated with cyclophosphamide alone, as manifested by reduction in the ovarian weight, decrease in the number of follicles and a fall in serum oestradiol, there was no elevation in serum gonadotrophin levels. Similar results were reported by Jarrel *et al.* (1987*a*). This suggests a possible cytotoxic effect of cyclophosphamide on the hypothalamo-pituitary axis leading to a reduction in gonadotrophin secretion. Weight loss and chronic stress could be also involved in these phenomena (Frisch & McArther, 1974; Hulse *et al.*, 1982). Although the initial differentiation of somatic elements into follicular cells occurs even in the absence of circulating gonadotrophin, normal development of medium sized to large follicles depends on gonadotrophic stimulation (Hardy *et al.*, 1974; Lunenfeld *et al.*, 1975). Interference with the pituitary secretion could be an additional mechanism responsible for gonadal failure seen in these animals. Since no alterations were detected in the pituitary-gonadal axis in the group treated with the combination as compared to controls, it is feasible that the functional inhibition of the pituitary gonadotrophs produced by the LH-RH agonist, protects the hypophyseal cells against a possible damage inflicted by cyclophosphamide.

As expected, the animals treated with cyclophosphamide alone showed a marked decrease in their body weight (Ataya *et al.*, 1985; Jarrel *et al.*, 1987*a*), while the rats receiving only D-Trp$^6$-LH-RH exhibited a significant increase, which could be attributed to the pharmacological castration produced by the LH-RH agonists (Bokser *et al.*, 1989). The group treated with the combination also showed an augmentation in the body weight as compared to controls. This raises an intriguing possibility that other organs may have been protected against the toxic effects of cyclophosphamide. However, no differences were found in peripheral white blood cell count (WBC) between the groups treated with cyclophosphamide alone or in combination with D-Trp$^6$-LH-RH, the WBC of both groups being lower than in untreated controls (data not shown). This indicates that the analogue was unable to prevent the toxic effects of cyclophosphamide on the bone marrow. Ataya *et al.* (1988) showed that, in contrast to the ovary, the treatment with an LH-RH agonist did not inhibit mitotic activity in the duodenum, skeletal muscle or bone marrow. Further investigations are necessary in order to evaluate a possible systemic protection against the deleterious actions of cytotoxic drugs.

It was interesting that 3 months after the treatment, the groups receiving D-Trp$^6$-LH-RH microcapsules or the combination with cyclophosphamide exhibited a higher number of follicles per ovary than the control group. Hypophysectomy also retards the rate at which the oocytes disappear from the ovaries in mice (Jones & Krohn, 1961). This suggests that the inhibition of the gonadotrophin secretion induced by LH-RH agonists is also able to retard the normal process of progressive loss of oocytes caused by the natural mechanism of aging (Ataya *et al.*, 1989).

Whereas repeated administration of LH-RH agonists is required to reduce the levels of LH, FSH and sex steroids, an immediate inhibition can be obtained after the first injection of LH-RH antagonists (Bajusz *et al.*, 1988*a*, *b*; Schally *et al.*, 1980; Schally, 1989; Vickery, 1986). Recently, we have synthesised highly potent antagonists of LH-RH, free of side-effects in animals and humans (Bajusz *et al.*, 1988*a,b*; Gonzalez-Barcena *et al.*, 1989; Schally, 1989). In addition, we have developed prototypes of sustained delivery systems consisting of microcapsules of our LH-RH antagonist SB-75 (Csernus *et al.*, 1990). Since, in patients afflicted with a malignant disease, chemotherapy may have to be started soon after the diagnosis is made, LH-RH antagonists may be preferred over the agonists for induction of gonadal inhibition, considering the rapidity of their suppressive action (Karashima *et al.*, 1988; Schally *et al.*, 1980; Schally, 1989). The recent clinical availability of highly efficacious sustained delivery formulations of various LH-RH agonists and the development of new LH-RH antagonists free of side-effects, may make possible new approaches for the prevention of gonadal damage produced in patients subjected to cytotoxic chemotherapy.

The work described in this paper was supported by The National Institute of Health Grants CA 40003 and 40004 (to A.V.S.), by the Medical Research Service of the Veterans Administration, G. Harold and Leila Y. Mathers Foundation and US Cancer Research Council. This manuscript is dedicated to the memory of Dr Pablo Mileikovsky. We thank Martha Sampson and Ilona Janaky for their valuable experimental assistance. We also thank Dr K. Groot and S. Song for performing the hormone assays and the National Hormone and Pituitary Progam (NHPP) for the gift of materials used in radioimmunoassays.

### References

ABRAHAM, G.E. (1974). Radioimmunoassay of steroids in biological fluids. *Clin. Biochem.*, **71,** 193.

ATAYA, K.M., MCKANNA, J.A., WEINTRAUB, A.M., CLARK, M.R. & LE MAIRIE, W.J. (1985). A luteinizing hormone-releasing hormone agonist for the prevention of chemotherapy-induced ovarian follicular loss in rats. *Cancer Res.*, **45,** 3651.

ATAYA, K.M., PALMER, K.C., BLACKER, C.M., MOGHISSI, K.S. & MOHAMMAD, S.H. (1988). Inhibition of rat ovarian [$^3$H] thymidine uptake by luteinizing hormone-releasing hormone agonists: a possible mechanism for preventing damage by cytotoxic agents. *Cancer Res.*, **48,** 7552.

ATAYA, K., TADROS, M. & RAMAHI, A. (1989). Gonadotropin-releasing hormone agonist inhibits physiologic ovarian follicular loss in rats. *Acta Endocrinol.*, **121,** 55.

BAJUSZ, S., CSERNUS, V., JANAKY, T., BOKSER, L., FEKETE, M. & SCHALLY, A.V. (1988*a*). New antagonists of LH-RH: II. Inhibition and potentiation by closely related analogues. *Int. J. Peptide Protein Res.*, **32,** 425.

BAJUSZ, S., KOVACS, M., GAZDAG, M. & 6 others (1988*b*). Highly potent antagonists of luteinizing hormone-releasing hormone free of edematogenic effects. *Proc. Natl Acad. Sci. USA*, **86,** 1637.

BOKSER, L., ZALATNAI, A. & SCHALLY, A.V. (1989). Inhibition of pituitary-gonadal axis in mice by long-term administration of D-Trp-6-LH-RH microcapsules. *J. Reprod. Fert.*, **85,** 569.

CHAPMAN, M. & SUTCLIFFE, S.B. (1981). Protection of ovarian function by oral contraceptives in women receiving chemotherapy for Hodgkin's disease. *Blood*, **58,** 849.

CHAPMAN, R.M., SUTCLIFFE, S.B. & MAPLES, J.S. (1979). Cytotoxic-induced ovarian failure in women with Hodgkin's disease: I. hormone function. *JAMA*, **242,** 1877.

CSERNUS, V.J., SZENDE, B., GROOT, K., REDDING, T.W. & SCHALLY, A.V. (1990). Development of Radioimmunoassay for a potent luteinizing hormone-releasing hormone antagonist: Evaluation of serum levels after injection of [Ac-3-(2-naphthyl)-D-Ala$^1$, D-Phe(pCl)$^2$, 3-(3-pyridyl)-D-Ala$^3$, D-Cit$^6$, D-Ala$^{10}$] LHRH. *Arzneimittel-Forschung*, **40,** 111.

DA CUNHA, M.F., MEISTRICH, M.L. & NADER, S. (1987). Absence of testicular protection by a gonadotropin-releasing hormone analogue against cyclophosphamide-induced testicular cytotoxicity in mouse. *Cancer Res.*, **47,** 1093.

DAMEWOOD, M.D. & GROCHOW, L.B. (1986). Prospects of fertility after chemotherapy or radiation for neoplastic disease. *Fertil. Steril.*, **45,** 443.

DELIC, J.I., BUSH, C. & PECKHAM, M.J. (1986). Protection from procarbazine-induced damage of spermatogenesis in rat by androgens. *Cancer Res.*, **46,** 1909.

DELIC, J.I., HARWOOD, J.R. & STANLEY, J.A. (1987). Time dependence for the protective effect of androgen from procarbazine-induced damage to rat spermatogenesis. *Cancer Res.*, **47,** 1344.

DORNFELD, E., SLATER, D. & SCHEFFE, H. (1942). A method for accurate determination of volume and cell numbers in small organs. *Anat. Rec.*, **82,** 255.

FRISCH, R.E. & MCARTHUR, J.W. (1974). Menstrual cycles: fatness as a determinant of minimum weight for height necessary for their maintenance or onset. *Science*, **185,** 949.

GLODE, L.M., ROBINSON, J., GOULD, S.F., NETT, T.M. & MERRIL, D. (1982). Protection of spermatogenesis during chemotherapy. *Drugs Exp. Clin. Res.*, **8,** 367.

GONZALEZ-BARCENA, D., VADILLO-BUENFIL, M., GARCIA-PORCEL, E. & 4 others (1989). Inhibition of LH and FSH release in the hypergonadotrophic patients with new potent antagonists of LH-RH free of anaphylactoid reactions. Presented at the 71st Annual Meeting Endocrine Society, Seattle, WA, 21–24 June, 1989, p. 450, abstract no. 1712.

GOODPASTURE, J.C., BERGSTROM, K. & VICKERY, B.H. (1988). Potentiation of the gonadotoxicity of cytoxan in the dog by adjuvant treatment with a luteinizing hormone-releasing hormone agonist. *Cancer Res.*, **48,** 2174.

GRADISHAR, W.R. & SCHILSKY, R.L. (1988). Effects of cancer treatment on the reproductive system. *CRC Crit. Rev. Oncol. Hematol.*, **8,** 153.

HARDY, B., DANON, D. & ESHKOL, A. (1974). Ultrastructural changes in the ovaries of infant mice deprived of endogenous gonadotrophins and after substitution with FSH. *J. Reprod. Fertil.*, **36,** 345.

HIRSCHFIELD, A. & MIDGLEY, A. (1978). Morphometric analysis of follicular development in the rat. *Biol. Reprod.*, **19,** 597.

HULSE, G., COLEMAN, G., NICHOLAS, J. & GREENWOOD, K. (1982). Reversal of the anti-ovulatory action of stress in rats by prior administration of naloxone hydrochloride. *J. Reprod. Fertil.*, **66,** 451.

JARREL, J., YOUNG LAI, E.V., BARR, R., MCMAHON, A., BELBECK, L. & O'CONNELL, G. (1987*a*). Ovarian toxicity of cyclophosphamide alone and in combination with ovarian irradiation in the rat. *Cancer Res.*, **47,** 2340.

JARREL, J., YOUNG LAI, E.V., MCMAHON, A., BARR, R., O'CONNELL, G. & BELBECK, L. (1987*b*). Effects of ionizing radiation and pretreatment with [D-Leu$^6$-des Gly$^{10}$] luteinizing hormone-releasing hormone ethylamide on developing rat ovarian follicles. *Cancer Res.*, **47,** 5005.

JONES, E.C. & KROHN, P.L. (1961). The effect of hypophysectomy on age changes in the ovaries of mice. *J. Endocrinol.*, **21,** 497.

KARASHIMA, T., ZALATNAI, A. & SCHALLY, A.V. (1988). Protective effects of analogs of luteinizing hormone-releasing hormone against chemotherapy-induced testicular damage in rats. *Proc. Natl Acad. Sci. USA*, **85,** 2329.

LEWIS, R.W., DOWLING, K.J. & SCHALLY, A.V. (1985). D-Tryptophan-6 analog of luteinizing hormone releasing hormone as a protective agent against the testicular damage caused by cyclophosphamide in baboons. *Proc. Natl Acad. Sci. USA*, **82,** 2975.

LUNENFELD, B., KRAIEM, Z. & ESHKOL, A. (1975). The function of the growing follicle. *J. Reprod. Fertil.*, **45,** 567.

MASON-GARCIA, M., VIGH, S., COMARU-SCHALLY, A.M. & 4 others (1985). Radioimmunoassay for 6-D-Tryptophan analog of luteinizing hormone-releasing hormone: measurement of serum levels after long acting microcapsule formulation. *Proc. Natl Acad. Sci. USA*, **82,** 1547.

NISWENDER, G.D., MIDGLEY, A.R. JR, MONROE, S.E. & REICHERT, L.E. JR (1968). Radioimmunoassay for rat luteinizing hormone with antiovine LH serum and ovine LH-$^{131}$I. *Proc. Soc. Exp. Biol. Med.*, **128,** 807.

NSEYO, U.O., HUBEN, R.P., KLIOZE, S.S. & PONTES, E. (1985). Protection of germinal epithelium with luteinizing hormone-releasing hormone analogue. *J. Urol.*, **34,** 187.

REDDING, T.W. & SCHALLY, A.V. (1983). Inhibition of mammary tumor growth in rats and mice by administration of agonistic and antagonistic analogs of luteinizing hormone-releasing hormone. *Proc. Natl Acad. Sci. USA*, **80,** 1459.

RIVKEES, A.S. & CRAWFORD, J.D. (1988). The relationship of gonadal activity and chemotherapy-induced gonadal damage. *JAMA*, 259, 2123.

SAMAAN, N.A., DE ASIS, D.N., BUZDAR, A.U. & BLUMENSCHEIN, G.R (1978). Pituitary-ovarian function in breast cancer patients on adjuvant chemoimmunotherapy. *Cancer*, **41,** 2084.

SCHALLY, A.V. (1989). The use of LHRH analogs in gynecology and tumor therapy. In *General Gynecology*, Belfort, P., Pinotti, J.A. & Eskes, T.K.A.B. (eds) p. 3. Parthenon: Carnforth.

SCHALLY, A.V., COY, D.H. & ARIMURA, A. (1980). LH-RH agonists and antagonists. *Int. J. Gynaecol. Obstet.*, **18,** 318.

SCHALLY, A.V., PAZ-BOUZA, J.I., SCHLOSSER, J.V. & 4 others (1987). Protective effects of analogs of luteinizing hormone-releasing hormone against X-radiation-induced testicular damage in rats. *Proc. Natl Acad. Sci. USA*, **84,** 851.

VICKERY, B.H. (1986). Comparison of the potential for therapeutic utilities with gonadotrophin-releasing hormone agonists and antagonists. *Endocr. Rev.*, **7,** 115.

WANG, C., FRACP, R.P., CHAN, T.K. & TODD, D. (1980). Effect of combination chemotherapy on pituitary-gonadal function in patients with lymphoma and leukemia. *Cancer*, **45,** 2030.